# Factors predicting the instant effect of motor function after subthalamic nucleus deep brain stimulation in Parkinson’s disease

**DOI:** 10.1186/s40035-017-0084-6

**Published:** 2017-05-26

**Authors:** Xin-Ling Su, Xiao-Guang Luo, Hong Lv, Jun Wang, Yan Ren, Zhi-Yi He

**Affiliations:** 1 Department of Neurology, First Affiliated Hospital, China Medical University, China Medical University, 155 Nanjing North Street, Heping District, Shenyang, 110001 China; 2 Department of Neurosurgery, First Affiliated Hospital, China Medical University, China Medical University, Shenyang, China

**Keywords:** Parkinson’s disease, Deep brain stimulation, Subthalamic nucleus, Predictive factors, Levodopa responsiveness

## Abstract

**Background:**

Subthalamic nucleus deep brain stimulation (STN-DBS) is an effective treatment for Parkinson’s disease (PD), the predictive effect of levodopa responsiveness on surgical outcomes was confirmed by some studies, however there were different conclusions about that through long- and short-term follow-ups. We aimed to investigate the factors which influence the predictive value of levodopa responsiveness, and discover more predictive factors of surgical outcomes.

**Methods:**

Twenty-three PD patients underwent bilateral STN-DBS and completed our follow-up. Clinical evaluations were performed 1 week before and 3 months after surgery.

**Results:**

STN-DBS significantly improved motor function of PD patients after 3 months; preoperative levodopa responsiveness and disease subtype predicted the effect of DBS on motor function; gender, disease duration and duration of motor fluctuations modified the predictive effect of levodopa responsiveness on motor improvement; the duration of motor fluctuations and severity of preoperative motor symptoms modified the predictive effect of disease subtype on motor improvement.

**Conclusions:**

The intensity of levodopa responsiveness served as a predictor of motor improvement more accurately in female patients, patients with shorter disease duration or shorter motor fluctuations; PD patients with dominant axial symptoms benefit less from STN-DBS compared to those with limb-predominant symptoms, especially in their later disease stage.

## Background

Parkinson’s disease (PD) is the second most common neurodegenerative disorder and is characterized by progressive loss of dopaminergic neurons in the substantia nigra; the cardinal clinical motor symptoms include tremor, rigidity, bradykinesia and axial symptoms [1]. Levodopa replacement therapy is the standard treatment for PD but causes motor complications as the disease progresses [1]. Subsequently, bilateral deep brain stimulation (DBS) of the subthalamic nucleus (STN) is used for patients with drug-refractory tremor or patients with intolerable motor complications. The benefits of STN-DBS are incontestable and have been proven by short-term and long-term follow-up studies [2–7].

Factors related to the outcomes of STN-DBS are a major concern to clinicians who want to predict the surgical effects in patients before the operation. P.D. Charles et al. and Jianyu Li respectively reported that patients with better preoperative levodopa responsiveness and younger age showed greater effects of surgery after 3 months [8] and after a long-term follow-up [6]; Hae Yu Kim et al. [4] noted that preoperative levodopa responsiveness, gender, age and magnitude of the Hoehn and Yahr stage (H&Y stage) before surgery were predictive of surgical outcomes with more than 3 years of follow-up. However, Tsai et al. [9] discussed that preoperative levodopa responsiveness only led to consistent improvement in part III of the Unified Parkinson Disease Rating Scale (UPDRS III) 3 months after STN-DBS and this predictive effect did not exist after 18 months. They also reported that disease duration, severity or H&Y stage did not predict improvement from short- and long-term STN-DBS; this result was contrary to the findings of other studies [4, 8]. While questions remain regarding why there are differing correlations between levodopa responsiveness and surgical improvement in long-term follow-ups or compared to that of short-term follow-up, and whether there are unknown factors influencing the predictive effects of levodopa responsiveness on DBS outcomes. Additionally, no consensus exists regarding the above-mentioned predictors, and whether there are any other characteristics that can predict the effects of surgery yet to be confirmed.

In the present retrospective study, we confirmed the effects of bilateral STN-DBS with a 3-month follow-up. The 3 month was selected as a short-term follow-up because optimal surgical effects generally appear 3–6 months postoperatively [10] and most patients attained a relatively stable condition 3 months postoperatively [11]. Moreover, we aimed to identify other predictive factors for the effects of STN-DBS after 3 months, and it was worth emphasizing that we further performed stratified analyses to explore which factors modify the predictive effects of preoperative levodopa responsiveness and other analyzed predictors of postoperative motor improvement.

## Methods

### Patients

We studied 27 consecutive PD patients who underwent bilateral STN-DBS in the First Affiliated Hospital of China Medical University from November 2014 to November 2015, including 22 Medtronic DBS system and 5 PINS DBS system. All patients enrolled in the study have written informed consent, and the local ethics committee approved the study. The inclusion criteria for the study were as follows: 1) patients must were diagnosed as idiopathic Parkinson’s disease by movement disorder neurologists based on the UK PD Brain Bank Criteria [12]; 2) disease duration > = 4 years; 3) were effective to levodopa; 4) with motor fluctuations or wearing-off phenomenon; 5) can cooperate with our follow-up. Exclusion criteria including: 1) obvious complications after surgery, such as hemorrhage, serious infection; 2) with dementia or severe psychotic symptoms; 3) marked cerebral atrophy or other abnormities on MRI.

### Surgery

**Fig. 1:**
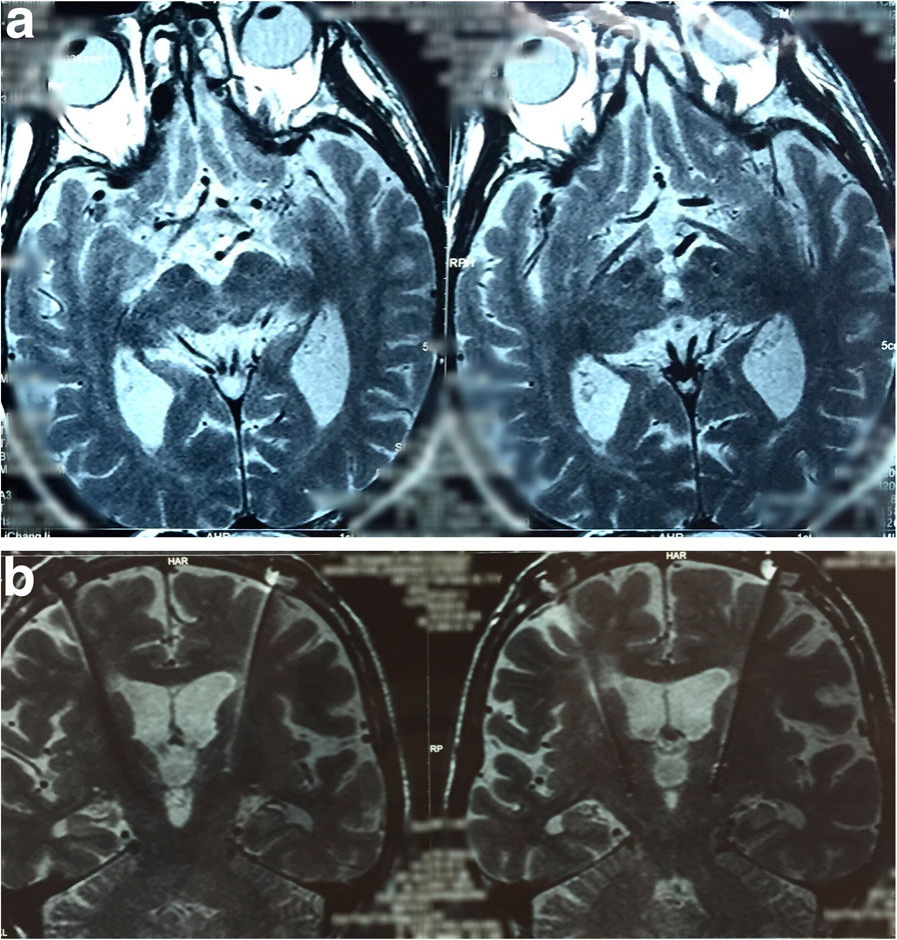
**a**. The lead location by the axial view of postoperative MRI. **b**. The lead location by the coronal view of postoperative MRI

We located the subthalamic nucleus by preoperative magnetic resonance imaging (MRI 3.0 T) with Leksell stereotactic frame, then compute accurate coordinates of the target by surgical-plan system, the subthalamic nuclei dorsolateral part was selected as the target; intraoperative microelectrode localization was taken use of the electrophysiological recordings, then we made a stimulation test to observe the reaction of patients to different voltages after electrode implantation, an impulse generator (IPG) was implanted subcutaneously when patients were under general anesthesia. All patients underwent MRI (1.5 T) postoperatively for the assessment of target location and surgical complications (Fig. 1). Patients followed the doctor’s advices to continue to take medicine and without stimulation settings temporarily.

### Programming

Approximately 1 month after surgery, we turned on the IPG when patients were totally at off-medication state (drug withdrawal more than 6 h), through repetitive tests, all the contacts were tested according to a standard protocol [13]. We set frequency at 130 Hz and pulse width at 60 us generally, the amplitude was progressively increased from 0 to 5–6 V with increments of 0.5–1.0 V or until side effects appeared. The optimal electrode contacts and voltage with the lowest threshold for inducing a beneficial results and the highest threshold for leading side effects were finally selected for chronic stimulation [7]. After setting up suitable parameters, we adjusted the dopaminergic medication based on the patient’s response to stimulation or just followed the preoperative medication plan. Then patients continued to observe at home, and accompanied with our telephone follow-up, patients came back to program when they feel uncomfortable, we adjusted the parameters on the basis of their symptoms, until up to a steady state.

### Assessments

**Fig. 2:**
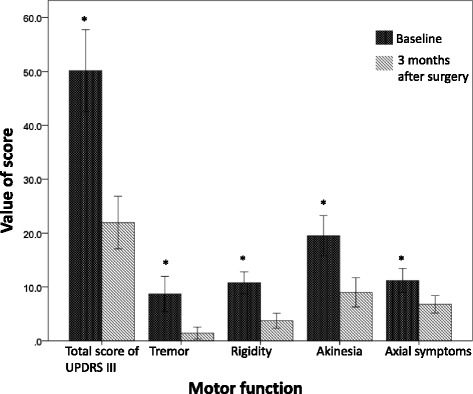
The comparison of motor function between baseline and 3 months after STN-DBS. Basal and postoperative scores (mean ± SE) for of UPDRS III and tremor, rigidity, akinesia, axial symptoms were showed in the diagram. Tremor obtained the most significant improvement and axial symptoms improved least

We collected the basic clinical information of all subjects before surgery. All patients with STN-DBS surgery were assessed by UPDRS III (item18–31) 1 week preoperatively and 3 months postoperatively, and to calculate the improvement of motor symptoms according to the assessment data, in other words, we measured the efficacy of STN-DBS on motor function through the change of UPDRS III scores before and after surgery (Fig. 2)

.

Pre-operation:

1. Acute levodopa challenge test: UPDRS III was evaluated when patients took no medicine for at least 12 h (usually overnight) which defined as “off-medication” or baseline state [14], and in the course of the maximal clinical benefit after administration of a dose of Madopar which was about 50 mg higher than the usual morning dose (“on-medication”) [15], the levodopa responsiveness which refers to the percentage improvement of levodopa challenge test was equivalent to (UPDRS III score of the baseline state - UPDRS III score of best state)/UPDRS III score of baseline*100%.
2. The akinesia scores include items 23–26 of UPDRS part III; the scores of axial symptoms include dysphonia, neck rigidity, arising from a chair, gait, and postural instability (items 18, 22 along with 27–30 of UPDRS III) [16].
3. The severity of Parkinsonism was evaluated by the scores of UPDRS III and H&Y stage in off-medication condition respectively.
4. LEDD refers to levodopa equivalent daily dose which was calculated as the dose of dopamine agonist plus levodopa and MAO-B inhibitor, according to the following formula: 100 mg Madopar = 1 mg pramipexole = 100 mg piribedil = 10 mg selegiline; each dose of levodopa was 25% more effective with entacapone. [7, 17].
5. Duration of motor fluctuations refers to the time from wearing-off symptoms emerge to the time of preoperative evaluation.
6. Based on the predominant motor features in daily living activities and motor scores of UPDRS, the disease subtype of an individual patient was classified as posture instability and gait difficulty (PIGD) [18] and limb-predominant symptoms (LPS). PIGD was defined as the scores of items 28–30, LPS was defined as the total scores of limb tremor, limb rigidity and akinesia (items 23–26). We used the ratio of the scores of LPS to the scores of PIGD to judge which subtype the patients belong to (>6 belong to LPS, <6 belong to PIGD, when the rate equal to or very close to 6, we grouped the patients according to their main complaints).

Post-operation:

Three months after STN-DBS, patients came to program the parameters, they must withdraw drugs for at least 6 h, when doctors finished the programming and patients reached to an ideal state, we assessed the score of UPDRS III at off-medication/on-stimulation condition.

The motor effects of STN-DBS was evaluated by the difference of UPDRSIII scores at off-medication state before and after operation, improvement rate was defined as follows: (preoperative UPDRS III score - postoperative UPDRS III score)/preoperative score *100%.

### Statistical analysis

The effects of bilateral STN-DBS on parkinsonian motor symptoms were evaluated using Wilcoxon signed rank test (Table 2). Then we performed univariate analysis and chose the variables whose *p* value was less than 0.1 or with assured clinical significance, next we did multivariate analysis after adjusting the potential confounders to estimate the independent relationship between postoperative motor function improvement and each related factors. When performed further stratified analysis, we determined the cut-off point of disease duration (<10 years, > = 10 years) based on that PD patients progressed to severe disability after about 10 years of the onset [19], the dividing line of duration of motor fluctuations (<=3 years, >3 years) was determined by reference to previous studies [16, 20, 21], and we chose the mean preoperative scores of UPDRS III as cut-off point (<=50, >50) since no clear cut-off has been confirmed. A *p* value less than 0.05 was regarded as statistically significant. All analyses were performed using Empower (R) (www.empowerstats.com, X&Y solutions, inc. Boston MA) and R (http://www.R-project.org).

## Results

### Demography and baseline characteristics of the PD patients

Four of the initial 27 patients in our study were lost during the follow-up. Finally, 23 patients (11 men and 12 women, 21 Medtronic and 2 PINS) with a mean (±SD) age of 50.4 ± 8.5 years at onset, 61.7 ± 8.3 years at the time of surgery and a mean disease duration of 11.3 ± 5.8 years were remained to complete the study, their mean duration of motor fluctuations was 5.4 ± 3.9 years, and mean score of UPDRSIII in off-medication condition was 50.2 ± 18.2. The baseline characteristics of the subjects were listed as Table 1. Additionally, the parameters at 3 months postoperatively including voltage (Left, Right), pulse width and frequency was 1.904 ± 0.45 V (L), 1.821 ± 0.39 V (R), 65 ± 9.45us, 138 ± 14.83 Hz respectively, and 18 of monopolar, 1 of bipolar and 4 of double cathode stimulation.

**Table 1:** Demography and baseline characteristics of study subjects

Characteristics	Value
Gender(male/female)	11/12
Age of onset, y	50.4 ± 8.5
Duration, y	11.3 ± 5.8
Age of surgery, y	61.7 ± 8.3
H&Y stage (off-med)	
Mild(<3)	9 (39.1%)
Severe(≥3)	14 (60.9%)
Levodopa responsiveness, %	62.5 ± 19.3
LEDD, mg/d	999.3 ± 516.7
Dyskinesia	
No	14 (60.9%)
Yes	9 (39.1%)
Motor fluctuations duration, y	5.4 ± 3.9
Disease subtype	
LPS	12(52.2%)
PIGD	11 (47.8%)
Baseline UPDRSIII scores (off-med)	50.2 ± 18.2

Data are expressed as numbers, with percentages in parentheses, or as means ± SE. *PIGD* posture instability and gait difficulty, *LPS* Limb-predominant symptoms; Baseline refers to “off-medication” state

### STN-DBS significantly improved the postoperative motor function of PD patients

Patients were followed up 3 months after operation. As illustrated in Table 2 and Fig. 2, motor function including total score of UPDRS III, scores of tremor, rigidity, akinesia and axial symptoms all demonstrated a significant improvement in “off-medication/on-stimulation” state compared with preoperative baseline state. The total score of UPDRSIII improved by 56% from 50.15 ± 18.19 at baseline to 21.94 ± 11.69 at 3 months (*p* < 0.001), tremor, rigidity, akinesia, and axial symptoms were ameliorated by 83% (*p* < 0.001), 66% (*p* < 0.001), 54% (*p* < 0.001) and 40% (*p* < 0.001) respectively. The postoperative levodopa equivalent daily doses decreased 20%, from 999.32 ± 516.69 mg at baseline to 797.52 ± 414.45 mg at 3 months (*p* = 0.006) (Table 2). No patient stopped taking medication.

**Table 2:** Comparison between baseline and 3 months postoperatively

Subscale	Range of possible scores	Preoperative baseline (*N* = 23)	3 months after surgery off-medication/on-stimulation (*N* = 23)	*P* value
Total UPDRSIII	0–108	50.15 ± 18.19	21.94 ± 11.69; 56%	<0.001**
Tremor	0–28	8.67 ± 7.85	1.44 ± 2.65; 83%	<0.001**
Rigidity	0–20	10.76 ± 4.82	3.71 ± 3.33; 66%	<0.001**
Akinesia	0–32	19.5 ± 8.95	8.98 ± 6.48; 54%	<0.001**
Axial symptoms	0–24	11.17 ± 5.35	6.76 ± 3.84; 40%	<0.001**
LEDD, mg		999.32 ± 516.69	797.52 ± 414.45; 20%	0.006*

**p < 0.01, *p < 0.05; Akinesia refers to the sum of items 23–26 of UPDRS III; Axial symptoms was defined as the sum of the following motor scores: item 18, 22 (rigidity of the neck), items 27–30 [16]

### Preoperative levodopa responsiveness and disease subtype influenced the effect of DBS operation on motor function

During the univariate analysis (Table 3), we selected the variables which the *p* value less than 0.1 or variables with definite clinical significance in other studies to make further analyses, included disease subtype and preoperative levodopa responsiveness. In the multivariate analysis (Table 3), after adjusting factors of gender, disease duration, subtype, baseline UPDRS III scores, dyskinesia, age of onset, age of surgery and duration of motor fluctuations, the results showed that preoperative levodopa responsiveness tended to be positively related to the improvement of motor function in “off-medication/on-stimulation” 3 months postoperatively (β = 0.9, 95% CI 0.1, 1.7, *p* = 0.055, on the verge of 0.05), it referred to that as each increase of 1% of preoperative levodopa responsiveness, the postoperative motor function improved as the similar amplitude of 0.9%. Additionally, there was a significant difference of postoperative motor improvement between PIGD group and LPS group in multivariate analysis after adjusting levodopa responsiveness, baseline UPDRS III scores, dyskinesia and age of surgery (β = −28.7, 95% CI -49.5, −8.0, *p* = 0.015), which indicated that the motor function in PIGD group was 28.7% less improved than LPS group.

**Table 3:** The correlations between various factors and postoperative improvement of motor function

Variables	Univariate analysis		Multivariate analysis	
	β	*P* value	β	*P* value
Gender (male/female)	7.1	0.451	10	0.364
Age of onset, y	−0.2	0.788	−0.3	0.719
Duration, y	−0.7	0.429	−0.3	0.852
Age of surgery, y	−0.5	0.397	−1	0.35
H&Y stage (Mild/Severe)	−6.4	0.503	5.5	0.74
Levodopa responsiveness, %	0.2	0.328	0.9	0.055
Dyskinesia(No/Yes)	−8.7	0.361	10.3	0.357
Motor fluctuations duration, y	−1	0.427	−1.7	0.495
Disease subtype (PIGD/LPS)	−17.2	0.057	−28.7	0.015*
Baseline UPDRSIII scores	0.3	0.331	0.3	0.421

*p < 0.05; *p* value which was close to 0.05 means a significant tendency; multivariate analyses of all factors adjusted the respective covariates

### Gender, disease duration and duration of motor fluctuations modified the effect of preoperative levodopa responsiveness on postoperative motor improvement

In order to discover whether some factors influenced the effect of levodopa responsiveness on motor results of STN-DBS, we further did stratified analysis, here we adjusted their related covariates respectively. The results in Table 4 demonstrated that gender, duration of motor fluctuations and disease duration exerted significant influence on the preoperative levodopa responsiveness-related postoperative motor function improvement.

**Table 4:** Factors that modify the predictive effects of levodopa responsiveness and disease subtype on motor improvement

Predictors	Stratification factors		β	95% CI	*P* value
Preoperative levodopa responsiveness	Gender	Female	1.1^a^	(0.3,1.9)	0.0294*
		Male	0.6^a^	(−0.4,1.6)	0.2901
	Disease duration, y	<10	2.0^a^	(1.1,2.9)	0.0021**
		> = 10	−0.1^a^	(−0.9,0.8)	0.8874
	Motor fluctuations duration, y	<=3	3.1^a^	(0.8,5.5)	0.0393*
		>3	0.5^a^	(−0.4,1.3)	0.3061
Disease subtype(PIGD/LPS)	Motor fluctuations duration, y	>3	−38.4^b^	(−67.1,−9.7)	0.039*
	Disease severity	>50	−41.1^b^	(−61.8,−20.4)	0.03*

^a^refers to the value of β means that as each increase of 1% of Levodopa responsiveness, the postoperative motor function improved as a certain amplitude; ^b^refers to the value of β means the difference of motor improvement of PIGD group compared to that of LPS group; **p < 0.01, *p < 0.05; Disease severity was measured by the baseline UPDRS scores of part III

Each 1% increment of preoperative levodopa responsiveness led to a 1.1% increase of motor improvement in female group (β = 1.1, 95% CI 0.3,1.9, *p* = 0.0294), while there was no statistical significance in male patients (Table 4); as each increase of 1% of preoperative levodopa responsiveness, the motor function improved 2.0% in patients with disease duration less than 10 years (β = 2.0, 95% CI 1.1,2.9, *p* = 0.0021), and no statistical significance in patients with longer disease duration (>10 years); each 1% increment of preoperative levodopa responsiveness led to a 3.1% increase of postoperative motor improvement in patients whose motor fluctuations appeared less than 3 years (β = 3.1, 95% CI 0.8,5.5, *p* = 0.0393), and no statistical significance in the others with motor fluctuations longer than 3 years (Table 4). Moreover, when stratified by factors such as age of onset, age of surgery and H&Y stage, we not found more other factors which can influenced the predicting power of Levodopa responsiveness.

### The duration of motor fluctuations and severity of preoperative motor symptoms modified the effect of disease subtype on postoperative motor improvement

The results of stratified analysis in Table 4 showed that more obvious difference was existed in patients with longer motor fluctuations (> = 3 years), in other words, in those late-stage operated patients with motor fluctuation longer than 3 years, the PIGD group was 38.4% less improved than LPS group (β = −38.4, 95% CI -67.1, −9.7, *p* = 0.039); additionally, in patients with more severe motor symptoms preoperatively whose baseline UPDRSIII score > 50, the PIGD group was 41.1% less improved than LPS group (β = −41.1, 95% CI -61.8, −20.4, *p* = 0.03); we found there was no difference of surgery effect between PIGD and LPS group when stratified by other factors.

## Discussion

Bilateral STN-DBS is widely used to treat PD and its distinct effects have been confirmed [2, 3, 6, 7, 11]. Compared with the “off-medication” condition prior to surgery, bilateral STN-DBS greatly improved the motor function of PD patients in “off-medication/on-stimulation” condition 3 months after surgery in our study. The total score of UPDRS III was significantly reduced by 56% of baseline. Scores of motor symptoms including tremor, rigidity, akinesia, and axial symptoms all decreased postoperatively, of these, tremor demonstrated the most improvement (83%) and axial symptoms showed minimal change (40%). In addition, postoperative medication dosage showed a marked decrease compared to preoperative dosage requirements.

The marked effects of STN-DBS after both short-term and long-term follow-up are well known, however, for clinicians, it is more important to evaluate the variables that may influence the clinical outcomes of surgery to optimize the timing of an operation and to predict the therapeutic effects of surgery as accurately as possible. Preoperative levodopa responsiveness is a well-established predictor of motor improvements after STN-DBS therapy [4, 6, 10, 22]. In our study, multivariate analyses demonstrated a positive and nearly statistically significant correlation between levodopa responsiveness and postoperative motor improvement (*p* = 0.055), where the *p* value may be attributed to the small sample size of our study. After adjusting for potential confounds in the analyses, stratifying for gender, disease duration and duration of motor fluctuations, a significant association was found between levodopa responsiveness and postoperative motor improvement.

The strong predicting effect of levodopa responsiveness generally suggests that the resolution of PD symptoms with DBS is more related to the degeneration of the dopaminergic system; greater involvement of other neurotransmitter systems, such as acetylcholine and noradrenaline, in the disease may contribute to the less predicting effect of levodopa responsiveness. Thus, our study demonstrates the three variables that may exert influence on the predictive power of levodopa responsiveness on postoperative motor improvements. The three significant variables are female gender, shorter disease duration, and shorter duration of motor fluctuations. This result not meant a great DBS response in these people or a poor surgical response in the male patients or patients with motor fluctuation more than 3 years or disease duration longer than 10 years, our point here is to tell which subset of patients can be more accurately predicted by preoperative levodopa responsiveness rather than tell the clues for worse DBS result.

In female patients, each 1% increment of preoperative levodopa responsiveness led to a 1.1% increase of postoperative motor improvement (*p* < 0.05), these data suggest that preoperative levodopa responsiveness may be a more accurate predictor for the outcomes of motor function after STN-DBS in female PD patients. We supposed this result may be related to the greater survival of dopaminergic neurons in women owing to the protective role of estrogens against the degeneration of dopaminergic neurons which had been suggested by primate model tests [23–25] and a clinical and epidemiological study [26]; in addition, estrogens were affirmed to prevent the dopamine depletion in studies using rodent PD models induced by 6-hydroxydopamine [27] and by 1-methyl-4-phenyl-1,2,3,6-tetrahydropyridine (MPTP) [28]. Accordingly, more dopaminergic neurons survived in female PD patient suggesting less disease severity, and DBS mainly ameliorated dopaminergic-related symptoms [29], thus, better responses to levodopa preoperatively predict greater improvement in motor function after STN-DBS in female PD patients.

The other two variables that influenced the predictive potency of levodopa responsiveness on postoperative motor improvement were shorter disease duration (< 10 years) and shorter duration of motor fluctuations (< 3 years) which both imply a DBS operation in the early stages of the disease, a so-called “early stimulated” condition. Similarly, early-stimulated groups also have fewer non-dopaminergic symptoms, including freezing of gait, postural instability, falls, or cognitive disorders; thus, parkinsonian symptoms in the early stages improve with levodopa supplementation or DBS as well. The biochemical mechanism in the late stages of PD, as suggested by a longer disease duration or longer time of motor fluctuation, was not only related to the loss of nigrostriatal dopaminergic neurons but also the participation of other non-dopaminergic systems, such as loss of noradrenaline in the locus coeruleus (LC), glutamatergic hyperactivity and loss of cholinergic pedunculopontine nucleus (PPN) neurons, as described by David Devos et al. [30]. In sum, we supposed that preoperative levodopa responsiveness serves as a predictor for DBS outcomes more precisely in early-stage PD patients than late-stage patients. Generally, the PD symptoms expected to be resolved with DBS are those responsive to levodopa supplementation, which suggests that DBS mainly exerts a dopaminergic-based effect. The two variables in our study that exerted greater influence on the relationship between levodopa responsiveness and postoperative motor improvement are related to better preservation of dopamine neurons or less severity of the disease.

Another variable predicting postoperative motor improvement in our study was the subtype of disease before surgery. We observed a significant difference in motor improvement between two predominant subtypes; the PIGD group showed poorer amelioration than the LPS group (*p* < 0.05), in other words, patients with dominant symptoms of LPS preoperatively gained greater motor function improvements than those in the PIGD group. After further stratified analyses, we found this difference between the two subtypes was notable in patients with longer duration of motor fluctuations (*p* < 0.05) and in patients with severe motor symptoms before surgery (*p* < 0.05); these findings suggest that PIGD patients at a late stage of PD would benefit less from the operation than LPS patients and an early recommendation for the operation for such subtype would be desirable. Given that motor function improvement with DBS manifests primarily in dopaminergic-related symptoms, it is conceivable to understand this result since for PIGD patients, the major symptoms such as falling, balancing dysfunction and gait disorders are axial symptoms, which are not completely alleviated by levodopa but also much related to non-dopamineric neurotransmitters [29, 31] and are involved in later stages of the disease [30–32].

One major shortcoming of our study was the small sample size of 23 patients, which decreased the *p* value of our statistical results. Other factors related to the surgical outcomes and the predictors reported in other studies may be confirmed through the statistical analysis of a larger patient population. Another limitation of this study was that we collected UPDRS III data but not UPDRS II and IV data, so other patient aspects were not evaluated. We expect that future studies with larger patient populations will confirm these findings.

## Conclusions

The intensity of preoperative levodopa responsiveness served as a predictor of motor improvement more accurately in female patients, and patients with short disease duration or shorter motor fluctuations. Patients with dominant axial symptoms as PIGD ones benefit less from STN-DBS compared to those with limb-predominant symptoms, especially in their later disease stage or with more severe motor symptoms before surgery. So it is natural and reasonable to recommend the operation to levodopa-responsive patients especially the female, patients in early stage of disease and PIGD patients at their early stage.

## References

[1] Lang AE, Lozano AM (1998). Parkinson’s disease. First of two parts. New Engl J Med.

[2] Tao Y, Liang G (2015). Effect of subthalamic nuclei electrical stimulation in the treatment of Parkinson's disease. Cell Biochem Biophys.

[3] Chiou SM, Lin YC, Huang HM (2015). One-year outcome of bilateral Subthalamic stimulation in Parkinson disease: an eastern experience. World Neurosurg.

[4] Kim HY, Chang WS, Kang DW, Sohn YH, Lee MS, Chang JW (2013). Factors related to outcomes of subthalamic deep brain stimulation in Parkinson's disease. J Korean Neurosurg Soc.

[5] Tir M, Devos D, Blond S, Touzet G, Reyns N, Duhamel A, Cottencin O, Dujardin K, Cassim F, Destée A, Defebvre L, Krystkowiak P (2007). Exhaustive, one-year follow-up of subthalamic nucleus deep brain stimulation in a large, single-center cohort of parkinsonian patients. Neurosurgery.

[6] Li J, Zhang Y, Li Y (2015). Long-term follow-up of bilateral subthalamic nucleus stimulation in Chinese Parkinson's disease patients. Br J Neurosurg.

[7] Jiang LL, Liu JL, Fu XL, Xian WB, Gu J, Liu YM, Ye J, Chen J, Qian H, Xu SH (2015). Long-term efficacy of Subthalamic nucleus deep brain stimulation in Parkinson's disease: a 5-year follow-up study in China. Chin Med J.

[8] Charles PD, Blercom NV, Krack P, Lee SL, Xie J, Besson G, Benabid A-L, Pollak P (2002). Predictors of effective bilateral subthalamic nucleusstimulation for PD. Neurology.

[9] Tsai ST, Lin SH, Chou YC, Pan YH, Hung HY, Li CW, Lin SZ, Chen SY (2009). Prognostic factors of subthalamic stimulation in Parkinson's disease: a comparative study between short- and long-term effects. Stereotact Funct Neurosurg.

[10] Bronstein JM, Tagliati M, Alterman RL, Lozano AM, Volkmann J, Stefani A, Horak FB, Okun MS, Foote KD, Krack P (2011). Deep brain stimulation for Parkinson disease: an expert consensus and review of key issues. Arch Neurol.

[11] Limousin P, Krack P, Pollak P (1998). Electrical stimulation of the Subthalamic nucleus in advanced Parkinson’s disease. N Engl J Med.

[12] Hughes AJDS, Kilford L, Lees AJ (1992). Accuracy of clinical diagnosis of idiopathic Parkinson’s disease: a clinicopathological study of 100 cases. J Neurol Neurosurg Psychiatry.

[13] Volkmann J, Moro E, Pahwa R (2006). Basic algorithms for the programming of deep brain stimulation in Parkinson's disease. Mov Disord.

[14] Langston JW, Widner H, Goetz CG, Brooks D, Fahn S, Freeman T, Watts R (1992). Core assessment program for intracerebral transplantations (CAPIT). Mov Disord.

[15] Albanese A, Bonuccelli U, Brefel C, Chaudhuri KR, FRCP CC, Eichhorn T, Melamed E, Pollak P, Laar TV, Zappia M (2001). Consensus statement on the role of acute dopaminergicchallenge in Parkinson s disease. Mov Disord.

[16] Merola A, Romagnolo A, Bernardini A, Rizzi L, Artusi CA, Lanotte M, Rizzone MG, Zibetti M, Lopiano L (2015). Earlier versus later subthalamic deep brain stimulation in Parkinson's disease. Parkinsonism Relat Disord.

[17] Tomlinson CL, Stowe R, Patel S, Rick C, Gray R, Clarke CE (2010). Systematic review of levodopa dose equivalency reporting in Parkinson’s disease. Mov Disord.

[18] Jankovic J, McDermott M, Gauthier S, Goetz C, Golbe L, Huber S, Koller W, Olanow C, Shoulson I, Stern M (1990). Variable expression of Parkinson s disease: a base-line analysis of the DATATOP cohort. Neurology.

[19] Maetzler W, Liepelt I, Berg D (2009). Progression of Parkinson's disease in the clinical phase potential markers. Lancet Neurol.

[20] Mestre TA, Espay AJ, Marras C, Eckman MH, Pollak P, Lang AE (2014). Subthalamic nucleus-deep brain stimulation for early motor complications in Parkinson's disease-the EARLYSTIM trial: early is not always better. Mov Disord.

[21] Deuschl G, Schupbach M, Knudsen K, Pinsker MO, Cornu P, Rau J, Agid Y, Schade-Brittinger C (2013). Stimulation of the subthalamic nucleus at an earlier disease stage of Parkinson's disease: concept and standards of the EARLYSTIM-study. Parkinsonism Relat Disord.

[22] Kleiner-Fisman G, Herzog J, Fisman DN, Tamma F, Lyons KE, Pahwa R, Lang AE, Deuschl G (2006). Subthalamic nucleus deep brain stimulation: summary and meta-analysis of outcomes. Mov Disord.

[23] Leranth CRR, Elsworth JD, Naftolin F, Horvath TL, Redmond DE (2000). Estrogen is essential for maintaining nigrostriatal dopamine neurons in primates: implications for Parkinson's disease and memory. J Neurosci.

[24] Henderson VW (2006). The neurology of menopause. Neurologist.

[25] Gillies GE, Murray HE, Dexter D, McArthur S (2004). Sex dimorphisms in the neuroprotective effects of estrogen in an animal model of Parkinson's disease. Pharmacol Biochem Behav.

[26] Yadav R, Shukla G, Goyal V, Singh S, Behari M (2012). A case control study of women with Parkinson's disease and their fertility characteristics. J Neurol Sci.

[27] Gerlach M, Riederer P (2006). Animal models of Parkinson’s disease: an empirical comparison with the phenomenology of the disease in man. J Neural Transm.

[28] Dluzen DE, McDermott JL, Liu B (1996). Estrogen alters MPTP-induced neurotoxicity in female mice: effects on striatal dopamine concentrations and release. J Neurochem.

[29] Bejjani BP, Gervais D, Arnulf I, Papadopoulos S, Demeret S, Bonnet AM, Cornu P, Damier P, Agid Y (2000). Axial parkinsonian symptoms can be improved:the role of levodopa and bilateral subthalamic stimulation. J Neurol Neurosurg Psychiatry.

[30] Devos D, Defebvre L, Bordet R (2010). Dopaminergic and non-dopaminergic pharmacological hypotheses for gait disorders in Parkinson’s disease. Fundam Clin Pharmacol.

[31] Fasano A, Aquino CC, Krauss JK, Honey CR, Bloem BR (2015). Axial disability and deep brain stimulation in patients with Parkinson disease. Nat Rev Neurol.

[32] Coelho M, Ferreira JJ (2012). Late-stage Parkinson disease. Nat Rev Neurol.

